# Meta-analysis of RNA interaction profiles of RNA-binding protein using the RBPInper tool

**DOI:** 10.1093/bioadv/vbae127

**Published:** 2024-08-26

**Authors:** Joseph A Cogan, Natalia Benova, Rene Kuklinkova, James R Boyne, Chinedu A Anene

**Affiliations:** School of Biological Sciences, University of Huddersfield, Huddersfield, HD1 3DH, United Kingdom; School of Molecular and Cellular Biology, University of Leeds, Leeds, LS2 9JT, United Kingdom; Centre for Biomedical Science Research, School of Health, Leeds Beckett University, Leeds, LS1 3HE, United Kingdom; Centre for Biomedical Science Research, School of Health, Leeds Beckett University, Leeds, LS1 3HE, United Kingdom; Centre for Biomedical Science Research, School of Health, Leeds Beckett University, Leeds, LS1 3HE, United Kingdom; Centre for Biomedical Science Research, School of Health, Leeds Beckett University, Leeds, LS1 3HE, United Kingdom; Centre for Cancer Genomics and Computation Biology, Barts Cancer Institute, Queen Mary University of London, London, E1 4NS, United Kingdom

## Abstract

**Motivation:**

Recent RNA-centric experimental methods have significantly expanded our knowledge of proteins with known RNA-binding functions. However, the complete regulatory network and pathways for many of these RNA-binding proteins (RBPs) in different cellular contexts remain unknown. Although critical to understanding the role of RBPs in health and disease, experimentally mapping the RBP–RNA interactomes in every single context is an impossible task due the cost and manpower required. Additionally, identifying relevant RNAs bound by RBPs is challenging due to their diverse binding modes and function.

**Results:**

To address these challenges, we developed RBP interaction mapper RBPInper an integrative framework that discovers global RBP interactome using statistical data fusion. Experiments on splicing factor proline and glutamine rich (SFPQ) datasets revealed cogent global SFPQ interactome. Several biological processes associated with this interactome were previously linked with SFPQ function. Furthermore, we conducted tests using independent dataset to assess the transferability of the SFPQ interactome to another context. The results demonstrated robust utility in generating interactomes that transfers to unseen cellular context. Overall, RBPInper is a fast and user-friendly method that enables a systems-level understanding of RBP functions by integrating multiple molecular datasets. The tool is designed with a focus on simplicity, minimal dependencies, and straightforward input requirements. This intentional design aims to empower everyday biologists, making it easy for them to incorporate the tool into their research.

**Availability and implementation:**

The source code, documentation, and installation instructions as well as results for use case are freely available at https://github.com/AneneLab/RBPInper. A user can easily compile similar datasets for a target RBP.

## 1 Introduction

RNA-binding proteins (RBPs) ubiquitously regulate the fate and function of transcripts across all cellular processes (Hentze *et al.* 2018). RBP–RNA binding can occur through sequence-specific, structure-specific, and nonspecific mechanisms (Cook *et al.* 2010), and most RBPs function through transient recruitment of other components or formation of stable complexes. However, the nature of these interactions in different cell/tissue context is not fully characterized. The determination of the ‘RBP-interactome’, the transcriptome-wide set of transcripts bound by RBPs, is necessary to determine the processes that are regulated by those RBPs in different contexts. RBP–RNA immunoprecipitation coupled with sequencing, (RIP-Seq, eCLIP, PAR-CLIP) (Hafner *et al.* 2021) and knockdown/knockout of target RBP followed by RNA-sequencing (perturbation RNA-Seq) (Sternburg and Karginov 2020) have become popular methods to identify RBP-interactome. Tremendous progress has been achieved in the last decade in the profiling of hundreds of RBP interactions. Tens of hundreds of RIP-Seq, eCLIP-Seq, PAR-CLIP-Seq, and perturbation RNA-Seq has been performed on most RBPs and deposited on public repositories, suitable for mining to generate new insights into RBP functions.

While mining of existing RBP–RNA interaction datasets allows for cost effective generation of new biological insights and hypothesis, the analytical process is none trivial and poses challenges. First, individual profiles represent sample specific interactions, instead of global interactome. Although structural and sequence features of RBPs are conserved (Beckmann *et al.* 2015), the same RBP can have different interactomes in different cell states (Bi *et al.* 2021). Second, the experimental methods profile different aspects of the same interactome, leading to a strong correlation structure and dependence among the profiles (Chadeau‐Hyam *et al.* 2013). These challenges could be resolved if sample metadata are used to inform an integrative analysis. However, existing methods focus on visualization of input gene list rather than systematic data integration (Reimand *et al.* 2019). Moreover, no methods are available for metadata-directed integration of multi-omics datasets to account for unique features of RBP–RNA interactions profiles.

Here, we present RBPInper (https://github.com/AneneLab/RBPInper), which addresses these issues and generates robust global RBP interactomes. We demonstrate the utility of RBPInper through a use case on the integration of splicing factor proline and glutamine rich (SFPQ)–RNA interaction profiles. It generates a validated and robust global SFPQ interactome, revealing both novel and known components of SFPQ function.

## 2 Methods

### 2.1 RBPInper framework

**Figure 1.**
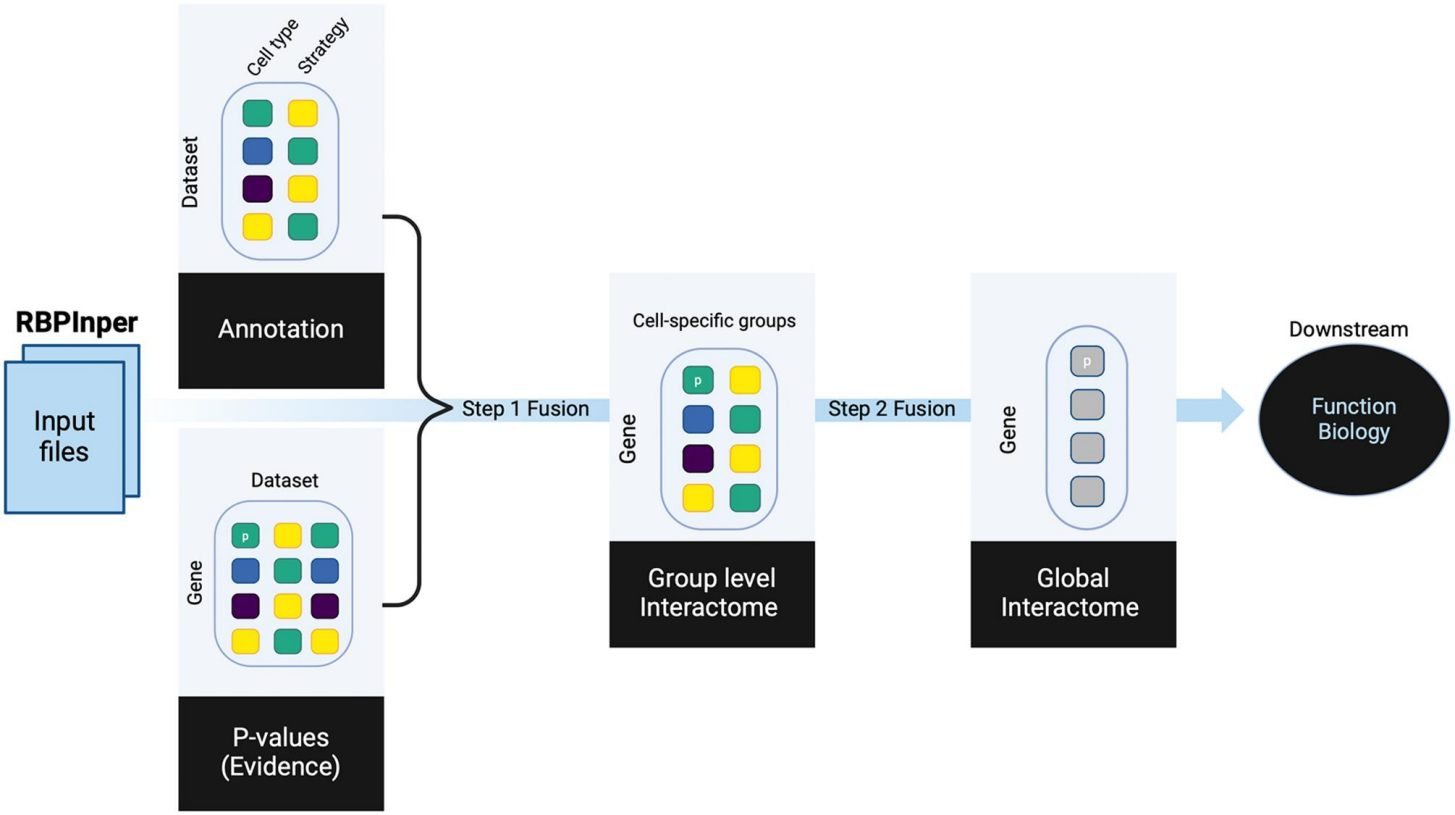
Overview of the RBPInper framework. RBPInper uses a meta-analysis approach to provide insight of RBP function. Evidence from multiple experimental datasets is directed through a *P*-value combination pipeline comprising two steps: (Step 1) Cell group-specific *P*-value combination and (Step 2) global *P*-value combination, to produce a single gene-wise *P*-value, and deduce the global RBP interactome.

RBPInper is an R tool that flexibly integrates the activities of an RBP from multiple experimental strategies (e.g. RNA-IP, eCLIP, and RNA-Seq) (Fig. 1). It takes two inputs: (i) a matrix of *P*-values with genes in rows and dataset evidence in columns, and (ii) a matrix of dataset information with ID in rows and annotations in columns (at least experimental strategy and the cell type). To integrate multiple sources of evidence, a combined *P*-value is computed for each gene using a twostep meta-analysis approach, resulting in a robust RBP–mRNA interaction gene list. First, the cell group-specific gene list is computed by merging all *P*-values of a given gene for each cell type into a combined *P*-value using the Bonferroni method (Goeman and Solari 2014, Cinar and Viechtbauer 2022). The assumption behind this approach is that *P*-values for the same cell type for the same RBP is correlated across the experimental strategies, thus, dependent. Ignoring this type of dependence when combining *P*-values can inflate the type I error rates (Alves and Yu 2014). Second, the global gene list is computed by combining all cell group-specific gene *P*-values using the Fisher’s combined probability test that accounts for the independence across the different cell types (Fisher 1970). The integrated list of Bonferroni *P*-values (cell group-specific) or Fisher *P*-values (global) are then individually corrected (using BH method) for multiple testing and filtered using a standard threshold (adjusted *P* < .05 default) to reflect current practices. However, the user can change the significance threshold at run time using the ‘*P*’ optional argument. Note, we set missing values such as the absence of a peak to *P*-value = 1.

We also include optional arguments in the function call to specify whether the *P*-values should be integrated with alternative methods [i.e. cell group-specific—dependence assumed: Bonferroni (default) or Harmonic mean (Wilson 2019), and global—independence assumed: Fisher (default) or Binomial (Wilkinson 1951)]. Furthermore, two outputs are provided; (i) global gene list, the default using all *P*-values, and (ii) cell type-specific gene list, which is derived by limiting the *P*-values to specific cell types. The RBPInper R package can be obtained through GitHub (https://github.com/AneneLab/RBPInper).

Depending on the experimental question, a user may wish to restrict the contribution of specific methods in the global gene list. Thus, we implement an optional penalty argument whereby all the data points for a restricted method are combined into one evidence (First step) and directly added to the second step with the rest of the data as described above. This argument ensures that the indicated method has marginal contribution to the final interactor call.

### 2.2 Pre-processing of bed peak files

To derive gene level *P*-value evidence for RNA-IP, eCLIP, and PAR-CLIP datasets we implemented an addon module ‘prebed()’ to prepare peak evidence. Given a standard bed file and a genome annotation file (gff), it assigns gene names to each peak based on gene coordinates. Then, we summarize the peak *P*-values by gene using the Bonferroni correction method (Goeman and Solari 2014). We include optional parameter to use other feature types (e.g. exon, transcript, CDS). Note, other standard bioinformatics analysis were conducted using our established pipeline (Anene *et al.* 2021) and described in the supplementary file.

### 2.3 Systematic mining of dataset and known SFPQ interactors

To demonstrate the use of RBPInper and evaluate its performance, we compiled a wide range of dataset on SFPQ RNA and DNA binding activity and function from Feingold *et al.* (2004), Yamazaki *et al.* (2018), Bi *et al.* (2021), Klotz-Noack *et al.* (2020), and Stagsted *et al.* (2021) (Supplementary Table S1).

For known interactors of SFPQ in existing literature, the RISMed R package was used to retrieve PubMed articles with cooccurring instances of SFPQ or aliases and other genes in titles and abstracts. The retrieved gene symbols were then mapped to their corresponding Ensembl IDs using the ‘mapIds()’ function from the ‘org.Hs.eg.db’ R package. The data mining approach is automated and included in the RBPInper package for user to apply on their own datasets.

### 2.4 RNA-Seq analysis

Raw reads were filtered to remove the adaptors and the low-quality reads (Q < 20) using Trimmomatic (Bolger *et al.* 2014). Filtered reads were aligned to the human reference genome GRCh38/hg38 assembly using HISAT2 (v2.1.0) in default settings (Kim *et al.* 2015). For SFPQ knockdown datasets, the counts in different genomic features were generated using HTSeq (v0.11.1) (Anders *et al.* 2015) on human GRCh38 reference annotation (GENCODE Release 32). Differential expression analyses between two groups were performed using the limma R package.

### 2.5 Simulation studies

To evaluate how well RBPInper estimates specific RBP interactome and its comparison to interactors derived independently from each dataset (herein called Union), we created pseudo-simulated table of *P*-value evidence, like previously reported approach (Chen 2011, Yoon *et al.* 2021). We based our simulations on real datasets to ensure it reflects the expected performance in real use cases. Using our compiled SFPQ dataset (*n* = 9) we randomly shuffled (10%, 20%, 30%, 40% and 50%) of the *P*-values of data sources to create random associations (technical noise). For each percentage, all combinations of the data sources were independently shuffled creating a large dataset for evaluation. Next, we derived SFPQ interactors using either RBPInper or Union. Then, we calculated the specificity of the calls in the shuffled data using the genes derived from the original unshuffled data as the expected interactome [Equation (1)].

(1) $$\mathrm{Specificity}=\;\frac{\mathrm{True}\;\mathrm{Negative}}{\mathrm{True}\;\mathrm{Negative}+\mathrm{False}\;\mathrm{Positives}}$$

## 3 Application

### 3.1 Transcriptome-wide discovery of the SFPQ–RNA interactome

**Figure 2.**
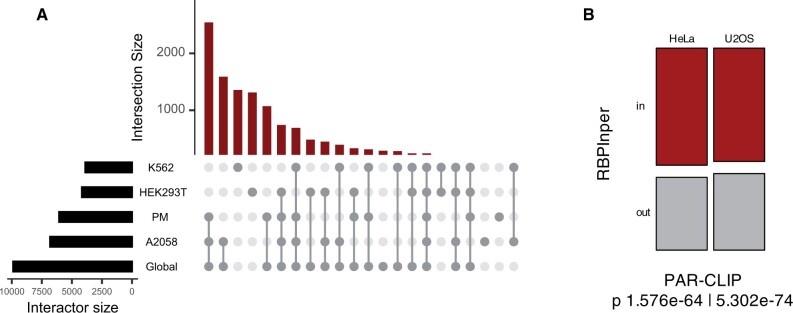
Robust discovery of RBP-interactomes. (A) Upset plot of generated hit count per cell group SFPQ interactome compared with RBPInper global SFPQ interactome. (B) Association of PAR-CLIP enriched genes and RBPInper derived SFPQ interactome stratified by cell line (GSE113349; HeLa, U2OS). Chi-squared (*P*) tests of independence are shown below panels. ‘in’ and ‘out’ represent genes overlapping or not overlapping with RBPInper calls, respectively.

To demonstrate the utility of RBPInper and evaluate its performance, we focused on a multi-functional RBP. Specifically, SFPQ interacts with a spectrum of RNAs partners to regulate many cellular processes such as transcription, DNA damage repair, and paraspeckle formation (Emili *et al.* 2002, Bladen *et al.* 2005, de Silva *et al.* 2019). Dysregulation of SFPQ interactions has been implicated in the aetiology of neurodegenerative diseases and a range of cancers. However, much of the interactions underpinning its physiological and pathological functions remains unknown. Thus, integrated analysis of its RNA activity profiles should enrich for both known and novel global interactors and biological processes. Note that existing SFPQ studies focused on single cell type or disease context, thus could not fully map the interactome. To this end, we collected two RIP-Seq, five perturbation RNA-Seq, and two eCLIP profiles from publicly available datasets (Supplementary Table S1) and applied our framework to derive global SFPQ interactome. We derived a 9884 gene interactome that was strongly enriched across the cell groups (Fig. 2A, Supplementary Table S2). To validate the genes, we obtained an independent pre-processed dataset of SFPQ PAR-CLIP of two cell lines (U2OS and HeLa) that were not covered in the integrated set (GSE113349) (Yamazaki *et al.* 2018). We extracted the genes associated with peaks and evaluated the overlap with the RBPInper derived interactome. We found that 60% (4575/7665, in U2OS) and 62% (3759/6097, in HeLa) of PAR-CLIP associated genes overlap with the integrated interactome, which is significantly higher than random chance (chi-squared *P* 1.576e-64: HeLa and *P* 5.302e-74: U2OS, Fig. 2B).

To further assess whether the RBPInper output captured known SFPQ interactors, we first defined known SFPQ interactions as genes previously associated with SFPQ in the literature (see Supplementary Methods). The known interactors also had significant overlap with RBPInper derived interactome (chi-squared *P* < 2.2e-16). These observations show that RBPInper extracts cognate RBP interactome by leveraging the combined power of the integrated experimental methods.

**Figure 3.**
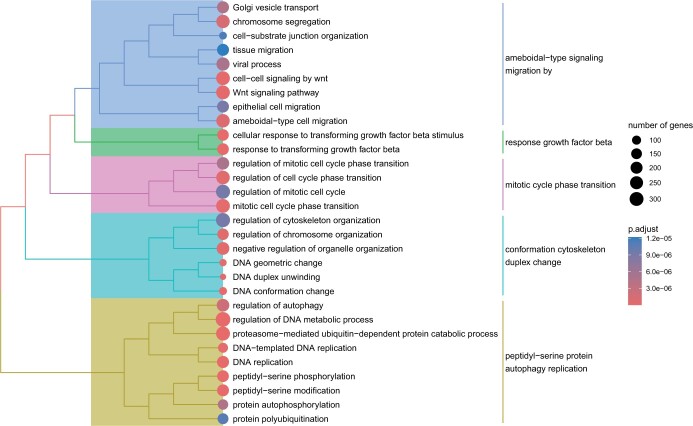
Enriched biological processes associated with SFPQ interactomes. Functional grouping tree diagram of enriched biological processes associated with global SFPQ interactome. Number of genes, total number of genes associated with given GO term. ‘*P*.adjust’, adjusted *P*-values by FDR.

We next evaluated the biological processes associated with the interactors using gene ontology analysis. We observed that SFPQ interactors are associated with a wide range of biological processes, including cell cycle, chromatin organization, cell-cell communication, biomolecule transport (Fig. 3, Supplementary Table S3), consistent with the multifunctional role of SFPQ signalling. Several of the enriched ontologies, such as heterochromatin formation and WNT signalling has not been previously linked to SFPQ (Supplementary Table S3), confirming that RBPInper provide new biological insight for understanding the function of RBPs.

**Figure 4.**
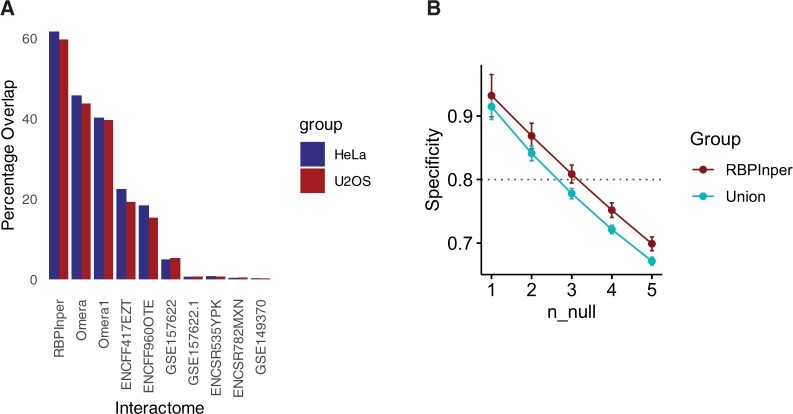
RBPInper exhibit robust RBP-interactome extraction capabilities. (A) Bar plot of percentage overlap between PAR-CLIP SFPQ enriched genes and RBPInper global SFPQ interactome or individual interactome datasets. (B) Specificity curve of the simulated RBP interactome recovered from the analysis of 763 simulated table of *P*-value evidence with known levels of random noise. n_null represents the number of randomly shuffled *P*-value columns corresponding to the percentage noise signals (1 = 10%, 2 = 20%, 3 = 30%, 4 = 40% and 5 = 50%). Union is the combined set of genes called from each data individually.

To assess the performance of the global integration approach against the individual samples, we called interactors for each profile individually using adjusted *P* < 0.05. Interrogating the overlap between interactors across the profiles and the independent PAR-CLIP data revealed surprisingly high overlap compared to using individual samples (Fig. 4A). RBPInper had the highest average overlap percentage (61%) compared to Omera (45%), Omera1 (40%), ENCFF417EZT (21%), ENCFF960OTE (17%), and GSE157622 (5%) (Fig. 4A). Although samples ENCSR535YPK, ENCSR782MXN, GSE149370, and GSE157622.1 had an average overlap of <1%, this is potentially due to fewer interactor calls (Fig. 4A). The high overlap of RBPInper is due to the robustness of the two-step meta-analysis integration (see Section 2).

Finally, to assess the performance of the RBPInper calls against different levels of technical noise and to compare it with union of the independently derived genes from above, we simulated random *P*-values with specific noise signals (10%–50% noise level), as described in the simulation studies. Then, for each simulated table of *P*-values (RBPInper or Union, see Section 2), we calculated the specificity of the derived genes overlapping the expected signals. Our results revealed that RBPInper has better specificity across all the noise levels compared to the union of the independently derived genes (Fig. 4B). The robust specificity of RBPInper results is due to the two-step meta-analysis core that combines the individual *P*-value evidence into a single consensus *P*-value (see Section 2). As expected, RBPInper’s ability to extract highly specific interactome is negatively associated with the level of technical noise (Fig. 4B). These results suggest that the quality of the interactome is dependent on the level of technical noise (e.g. incorrect peak identification due low quality of RNA) in the *P*-value estimations. On average, RBPInper has >93% and 85% specificity at 10% and 20% noise, respectively (Fig. 4B, Supplementary Table S6). Previous studies indicated that RNA-Seq datasets have technical noise <10%, while the rest of the differences is explained by biology and experimental conditions (SEQC Consortium 2014, Robinson *et al.* 2015). Thus, we believe that RBPInper retains its estimation power when analysing current real sequencing datasets across various RBPs. Note that the *P*-value input of RBPInper is derived from well-established tools for peak calling or differential gene expression analysis that have very low levels of errors in their estimations (Rapaport *et al.* 2013, Jeon *et al.* 2020).

Previous studies show that some mis-regulated genes in RBP perturbation data are downstream effects rather than direct interactors. Thus, we analysed our compiled datasets using the optional penalty argument (pen = perturbation) to investigate this utility. We found no major differences compared to the default unpenalized integration. 95.38% (9512 out of 9972) of the total genes derived were shared by the two approaches (unpenalized versus perturbation RNA-Seq penalized), suggesting that our meta-analysis integration can control for data specific bias. Although the unpenalized approach derived more unique genes (372) compared to the penalized (*n* = 88), both sets of unique genes were found to have good overlap with the independent validation sets (21.5% in U20S and 19.6% in HeLa cells) compared to the penalized version (37.5% in U20S and 34.1% in HeLa cells). These observations suggest that RBPInper can effectively reduce the limitations of individual datasets using either active or passive techniques.

### 3.2 Genome-wide discovery of the SFPQ–DNA interactome

Several RBPs have been shown to function through DNA binding of target genes. RBPInper can integrate DNA binding profiles with knockdown RNA-Seq data to provide information about the DNA level interactomes. As an example, here we focus on the DNA interactome of SFPQ, which is critical in its function in DNA repair (Ha *et al.* 2011). We integrated 6 SFPQ ChIP-Seq dataset with five perturbation RNA-Seq data (Supplementary Table S1), as the effect of RBP–DNA interactions should be present in SFPQ perturbation RNA-Seq data. We derived 2073 gene DNA interactome of SFPQ (Supplementary Table S4), which is less than the number of RNA interactors and consistent with the mainly RNA-binding function of SFPQ (Knott *et al.* 2016). Interestingly, 2036 out of 2073 (98%) of the DNA interactors were also found in the RNA interactome, indicating that SFPQ regulate some genes at both RNA and DNA levels.

## 4 Discussion

Characterizing RBP regulatory networks in different cellular contexts is of great interest to many research groups. However, experimentally performing this task for every single context and RBP is prohibitively expensive. Mining existing RBP interaction datasets has become a popular approach to remedy this issue. Unfortunately, there are no existing tools that provide gene-level integration of these datasets to identify reliable interactomes of RBP that can transfer to unseen contexts. First, interpreting multiple gene lists has been addressed by various algorithms and web-based methods. These approaches primarily focus on visualization of a single source of data, rather than empirical data integration of multiple evidence (Reimand *et al.* 2019). Second, existing tools for multiple multi-omics data integration lack the options to integrate RNA-IP, eCLIP, or PAR-CLIP, extensively reviewed in Bersanelli *et al.* (2016) and Subramanian *et al.* (2020). Thus, deriving robust global RBP interactomes is challenging. The RBPInper framework addressed this gap by providing systematic meta-analysis of gene-level evidence that exhibits consistent performance across various validation analyses and has further invaluable features not found in existing tools. The core utilities of RBPInper are that it (i) leverages the combined strengths of the individual experimental strategies for profiling RBP–RNA interactions while minimizing their weakness, and (ii) provides a global RBP interactome that extends beyond the immediate cell types and context profiled. Moreover, our framework is inherently versatile, allowing users to add new cell types and data groups at run time. We demonstrated this function by integrating ChIP-Seq and perturbation RNA-Seq data to enable the discovery of SFPQ’s DNA interactome. This flexible approach is critical for robustly estimating RBP interactomes that extend to new cellular contexts and represents an important advance compared to current approaches that exclusively focus on a single sample or context. RBPs have a wide range of roles in regulation of RNA and their dysregulation is involved in the development and progression of many diseases including cardiovascular diseases, autoimmune diseases, neurodegenerative diseases and cancers (de Bruin *et al.* 2017, Gebauer *et al.* 2021, Schieweck *et al.* 2021). However, detailed mechanisms of their role in these diseases are still lacking. As such, RBPInper provides a robust platform for mechanistic characterization of RBP interactions and functions, which can facilitate the development of new biomarkers and therapeutic targets.

Though using an imbalanced number of profiles per cell type may inadvertently bias the global interactome, multiple sources per RBP can better capture the diversity in RBP interactomes across different cell states. Unlike one cell type or sample approach, we solved this problem by first calculating the meta values individually for each cell group (see Section 2) before generating the global meta value for the same gene in each cell group. Here, a future update may include additional techniques to correct cell type bias such as two-tier group integration, particularly as interaction profiles in other cells become available. We also incorporated the option to extract and use group estimates, which is crucial for applications requiring interactomes in specific cell types.

Benchmarking on the individual samples showed that although each sample-specific calls retained some overlap with the independent dataset, we saw high levels of none overlap genes in those. This performance issue is expected but impossible to track in actual use cases because of the known cell-type specific interactions of RBP and experimental noise present in each sample. Our systematic meta-analysis reduces this sample bias by averaging out the sample-specific signals. Indeed, we adequately identified the novel WNT signalling and/or heterochromatin functions of SFPQ. Here, RBPInper identified EZH2, the catalytic subunit of PRC2 as a global interactor of SFPQ (adjusted *P* < 4.1e-16, see Supplementary Table S2). This finding is intriguing because previous research has shown that reducing SFPQ levels promotes EZH2 exon 14 skipping in a non-cell-type specific manner (Chen *et al.* 2017). Functionally, this shorter isoform lacks histone methyltransferase activity, but functions as an inhibitor of full-length EZH2. Since PCR2-mediated methylation of histone H3 on lysine 9, 27, or 37 is crucial for both constitutive and facultative heterochromatin formation (Chantalat *et al.* 2011), SFPQ may regulate heterochromatin formation through EZH2 alternative splicing.

Moreover, in zebrafish model, the depletion of the cytoplasmic pool of SFPQ (i.e. the SFPQ–RNA interactions) in a zebrafish model hindered motoneuron differentiation partly by autonomously reducing Wnt signalling (Thomas-Jinu *et al.* 2017). Interestingly, reintroducing cytosolic SFPQ restored Wnt signalling-related transcripts, revealing a context-specific function in motor axons. It is worth noting that single sample or cell type analysis could not resolve such a function due to their sample-specific noise, further highlighting the utility of our method. Future work will allow for a more specific characterization of other functions and mechanisms through which SFPQ contributes to various diseases.

An important limitation of RBPInper framework is that it represents the aggregate evidence of the contributing profiles. Thus, if they have universally high levels of technical noise for a given RBP, then RBPInper will have a corresponding spurious global interactome estimation. However, RBPInper should be robust for most cases compared to the individual samples or using the union of the interactors derived independently from the datasets. Indeed, our simulation studies show that RBPInper integration retains good specificity even at high technical noise. This observation is consistent with previous studies showing that meta-analysis enables the detection of small effects that might be false negatives in the individual datasets (Choi *et al.* 2003). It also has better reproducibility and reliability compared with individual datasets (Hong *et al.* 2006). Therefore, RBPInper is an indispensable tool to interrogate multiple datasets for global RBAP interactomes.

To enable easy incorporation of RBPInper into new and existing pipelines for analysing RBP interactions, we implemented an object-oriented system in R, allowing the user to add, run and retrieve individual elements of the analysis (https://github.com/AneneLab/RBPInper).

## Supplementary Material

vbae127_Supplementary_Data

## Data Availability

The datasets were derived from sources in the public domain: GEO: GSE149370, GSE157622, GSE113349; Encode: ENCSR782MXN, ENCSR535YPK, ENCFF598JWW, ENCFF919CIS, ENCFF073RTN, ENCFF923EYZ, ENCFF737MUN, ENCFF319CEQ, ENCFF417EZT, ENCFF960OTE and Article: Bi *et al.* (2021). Processed versions underlying this article are available in https://github.com/AneneLab/RBPInper.

## References

[vbae127-B1] Alves G , Yu Y-K. Accuracy evaluation of the unified P-value from combining correlated P-values. PLoS One 2014;9:e91225. 24663491 10.1371/journal.pone.0091225 PMC3963868

[vbae127-B2] Anders S , Pyl PT, Huber W. HTSeq—a Python framework to work with high-throughput sequencing data. Bioinformatics 2015;31:166–9. 25260700 10.1093/bioinformatics/btu638 PMC4287950

[vbae127-B3] Anene CA , Khan F, Bewicke-Copley F et al ACSNI: an unsupervised machine-learning tool for prediction of tissue-specific pathway components using gene expression profiles. Patterns (N Y) 2021;2:100270. 34179848 10.1016/j.patter.2021.100270 PMC8212143

[vbae127-B4] Beckmann BM , Horos R, Fischer B et al The RNA-binding proteomes from yeast to man harbour conserved enigmRBPs. Nat Commun 2015;6:10127. 26632259 10.1038/ncomms10127 PMC4686815

[vbae127-B5] Bersanelli M , Mosca E, Remondini D et al Methods for the integration of multi-omics data: mathematical aspects. BMC Bioinformatics 2016;17:15–177. 26821531 10.1186/s12859-015-0857-9 PMC4959355

[vbae127-B6] Bi O , Anene C, Nsengimana J et al SFPQ promotes an oncogenic transcriptomic state in melanoma. Oncogene 2021;40:5192–203. 34218270 10.1038/s41388-021-01912-4 PMC8376646

[vbae127-B7] Bladen CL , Udayakumar D, Takeda Y et al Identification of the polypyrimidine tract binding protein-associated splicing factor· p54 (nrb) complex as a candidate DNA double-strand break rejoining factor. J Biol Chem 2005;280:5205–10. 15590677 10.1074/jbc.M412758200

[vbae127-B8] Bolger AM , Lohse M, Usadel B. Trimmomatic: a flexible trimmer for Illumina sequence data. Bioinformatics 2014;30:2114–20. 24695404 10.1093/bioinformatics/btu170 PMC4103590

[vbae127-B9] Chadeau‐Hyam M , Campanella G, Jombart T et al Deciphering the complex: methodological overview of statistical models to derive OMICS-based biomarkers. Environ Mol Mutagen 2013;54:542–57. 23918146 10.1002/em.21797

[vbae127-B10] Chantalat S , Depaux A, Héry P et al Histone H3 trimethylation at lysine 36 is associated with constitutive and facultative heterochromatin. Genome Res 2011;21:1426–37. 21803857 10.1101/gr.118091.110 PMC3166828

[vbae127-B11] Chen K , Xiao H, Zeng J et al Alternative splicing of EZH2 pre-mRNA by SF3B3 contributes to the tumorigenic potential of renal cancer. Clin Cancer Res 2017;23:3428–41. 27879367 10.1158/1078-0432.CCR-16-2020 PMC5440213

[vbae127-B12] Chen Z. Is the weighted z‐test the best method for combining probabilities from independent tests? J Evol Biol 2011;24:926–30. 21401770 10.1111/j.1420-9101.2010.02226.x

[vbae127-B13] Choi JK , Yu U, Kim S et al Combining multiple microarray studies and modeling interstudy variation. Bioinformatics 2003;19:i84–90. 12855442 10.1093/bioinformatics/btg1010

[vbae127-B14] Cinar O , Viechtbauer W. The poolr package for combining independent and dependent p values. J Stat Soft 2022;101:1–42.

[vbae127-B15] Cook KB , Kazan H, Zuberi K et al RBPDB: a database of RNA-binding specificities. Nucleic Acids Res 2010;39:D301–8. 21036867 10.1093/nar/gkq1069 PMC3013675

[vbae127-B16] de Bruin RG , Rabelink TJ, van Zonneveld AJ et al Emerging roles for RNA-binding proteins as effectors and regulators of cardiovascular disease. Eur Heart J 2017;38:1380–8. 28064149 10.1093/eurheartj/ehw567

[vbae127-B17] de Silva HC , Lin MZ, Phillips L et al IGFBP-3 interacts with NONO and SFPQ in PARP-dependent DNA damage repair in triple-negative breast cancer. Cell Mol Life Sci 2019;76:2015–30. 30725116 10.1007/s00018-019-03033-4 PMC11105386

[vbae127-B18] Emili A , Shales M, McCracken S et al Splicing and transcription-associated proteins PSF and p54nrb/nonO bind to the RNA polymerase II CTD. RNA 2002;8:1102–11. 12358429 10.1017/s1355838202025037 PMC1370324

[vbae127-B19] Feingold E , Good P, Guyer M et al The ENCODE (ENCyclopedia of DNA elements) project. Science 2004;306:636–40. 15499007 10.1126/science.1105136

[vbae127-B20] Fisher RA. Statistical methods for research workers. Biological monographs and manuals. Oliver and Boyd 1970;10:0050021702.

[vbae127-B21] Gebauer F , Schwarzl T, Valcárcel J et al RNA-binding proteins in human genetic disease. Nat Rev Genet 2021;22:185–98. 33235359 10.1038/s41576-020-00302-y

[vbae127-B22] Goeman JJ , Solari A. Multiple hypothesis testing in genomics. Stat Med 2014;33:1946–78. 24399688 10.1002/sim.6082

[vbae127-B23] Ha K , Takeda Y, Dynan WS. Sequences in PSF/SFPQ mediate radioresistance and recruitment of PSF/SFPQ-containing complexes to DNA damage sites in human cells. DNA Repair (Amst) 2011;10:252–9. 21144806 10.1016/j.dnarep.2010.11.009 PMC3046316

[vbae127-B24] Hafner M , Katsantoni M, Köster T et al CLIP and complementary methods. Nat Rev Methods Primer 2021;1:20.

[vbae127-B25] Hentze MW , Castello A, Schwarzl T et al A brave new world of RNA-binding proteins. Nat Rev Mol Cell Biol 2018;19:327–41. 29339797 10.1038/nrm.2017.130

[vbae127-B26] Hong F , Breitling R, McEntee CW et al RankProd: a bioconductor package for detecting differentially expressed genes in meta-analysis. Bioinformatics 2006;22:2825–7. 16982708 10.1093/bioinformatics/btl476

[vbae127-B27] Jeon H , Lee H, Kang B et al Comparative analysis of commonly used peak calling programs for ChIP-Seq analysis. Genomics Inform 2020;18:e42. 33412758 10.5808/GI.2020.18.4.e42 PMC7808876

[vbae127-B28] Kim D , Langmead B, Salzberg SL. HISAT: a fast spliced aligner with low memory requirements. Nat Methods 2015;12:357–60. 25751142 10.1038/nmeth.3317 PMC4655817

[vbae127-B29] Klotz-Noack K , Klinger B, Rivera M et al SFPQ depletion is synthetically lethal with BRAFV600E in colorectal cancer cells. Cell Rep 2020;32:108184. 32966782 10.1016/j.celrep.2020.108184

[vbae127-B30] Knott GJ , Bond CS, Fox AH. The DBHS proteins SFPQ, NONO and PSPC1: a multipurpose molecular scaffold. Nucleic Acids Res 2016;44:3989–4004. 27084935 10.1093/nar/gkw271 PMC4872119

[vbae127-B31] Rapaport F , Khanin R, Liang Y et al Comprehensive evaluation of differential gene expression analysis methods for RNA-seq data. Genome Biol 2013;14:R95. 24020486 10.1186/gb-2013-14-9-r95 PMC4054597

[vbae127-B32] Reimand J , Isserlin R, Voisin V et al Pathway enrichment analysis and visualization of omics data using g: Profiler, GSEA, Cytoscape and EnrichmentMap. Nat Protoc 2019;14:482–517. 30664679 10.1038/s41596-018-0103-9 PMC6607905

[vbae127-B33] Robinson DG , Wang JY, Storey JD. A nested parallel experiment demonstrates differences in intensity-dependence between RNA-seq and microarrays. Nucleic Acids Res 2015;43:e131. 26130709 10.1093/nar/gkv636 PMC4787771

[vbae127-B34] Schieweck R , Ninkovic J, Kiebler MA. RNA-binding proteins balance brain function in health and disease. Physiol Rev 2021;101:1309–70. 33000986 10.1152/physrev.00047.2019

[vbae127-B35] SEQC Consortium. A comprehensive assessment of RNA-seq accuracy, reproducibility and information content by the Sequencing Quality Control Consortium. Nat. Biotechnol 2014;32:903. 25150838 10.1038/nbt.2957 PMC4321899

[vbae127-B36] Stagsted LVW , O'Leary ET, Ebbesen KK et al The RNA-binding protein SFPQ preserves long-intron splicing and regulates circRNA biogenesis in mammals. Elife 2021;10:e63088. 33476259 10.7554/eLife.63088 PMC7819710

[vbae127-B37] Sternburg EL , Karginov FV. Global approaches in studying RNA-binding protein interaction networks. Trends Biochem Sci 2020;45:593–603. 32531229 10.1016/j.tibs.2020.03.005

[vbae127-B38] Subramanian I , Verma S, Kumar S et al Multi-omics data integration, interpretation, and its application. Bioinform Biol Insights 2020;14:1177932219899051. 32076369 10.1177/1177932219899051 PMC7003173

[vbae127-B39] Thomas-Jinu S , Gordon PM, Fielding T et al Non-nuclear Pool of splicing factor SFPQ regulates axonal transcripts required for normal motor development. Neuron 2017;94:322–36.e5. 28392072 10.1016/j.neuron.2017.03.026 PMC5405110

[vbae127-B40] Wilkinson B. A statistical consideration in psychological research. Psychol Bull 1951;48:156–8. 14834286 10.1037/h0059111

[vbae127-B41] Wilson DJ. The harmonic mean p-value for combining dependent tests. Proc Natl Acad Sci USA 2019;116:1195–200. 30610179 10.1073/pnas.1814092116 PMC6347718

[vbae127-B42] Yamazaki T , Souquere S, Chujo T et al Functional domains of NEAT1 architectural lncRNA induce paraspeckle assembly through phase separation. Mol Cell 2018;70:1038–53.e7. 29932899 10.1016/j.molcel.2018.05.019

[vbae127-B43] Yoon S , Baik B, Park T et al Powerful p-value combination methods to detect incomplete association. Sci Rep 2021;11:6980. 33772054 10.1038/s41598-021-86465-y PMC7997958

